# Mosaic Arrangement of the 5S rDNA in the Aquatic Plant *Landoltia punctata* (Lemnaceae)

**DOI:** 10.3389/fpls.2021.678689

**Published:** 2021-06-24

**Authors:** Guimin Chen, Anton Stepanenko, Nikolai Borisjuk

**Affiliations:** ^1^Jiangsu Key Laboratory for Eco-Agricultural Biotechnology Around Hongze Lake, School of Life Sciences, Huaiyin Normal University, Huai’an, China; ^2^Jiangsu Collaborative Innovation Centre of Regional Modern Agriculture & Environmental Protection, Huaiyin Normal University, Huai’an, China

**Keywords:** duckweed, 5S rRNA genes, gene organization, molecular evolution, *Landoltia punctata*

## Abstract

Duckweeds are a group of monocotyledonous aquatic plants in the Araceae superfamily, represented by 37 species divided into five genera. Duckweeds are the fastest growing flowering plants and are distributed around the globe; moreover, these plants have multiple applications, including biomass production, wastewater remediation, and making pharmaceutical proteins. Dotted duckweed (*Landoltia punctata*), the sole species in genus Landoltia, is one of the most resilient duckweed species. The ribosomal DNA (rDNA) encodes the RNA components of ribosomes and represents a significant part of plant genomes but has not been comprehensively studied in duckweeds. Here, we characterized the 5S rDNA genes in *L. punctata* by cloning and sequencing 25 PCR fragments containing the 5S rDNA repeats. No length variation was detected in the 5S rDNA gene sequence, whereas the nontranscribed spacer (NTS) varied from 151 to 524 bp. The NTS variants were grouped into two major classes, which differed both in nucleotide sequence and the type and arrangement of the spacer subrepeats. The dominant class I NTS, with a characteristic 12-bp TC-rich sequence present in 3–18 copies, was classified into four subclasses, whereas the minor class II NTS, with shorter, 9-bp nucleotide repeats, was represented by two identical sequences. In addition to these diverse subrepeats, class I and class II NTSs differed in their representation of cis-elements and the patterns of predicted G-quadruplex structures, which may influence the transcription of the 5S rDNA. Similar to related duckweed species in the genus Spirodela, *L. punctata* has a relatively low rDNA copy number, but in contrast to Spirodela and the majority of other plants, the arrangement of the 5S rDNA units demonstrated an unusual, heterogeneous pattern in *L. punctata*, as revealed by analyzing clones containing double 5S rDNA neighboring units. Our findings may further stimulate the research on the evolution of the plant rDNA and discussion of the molecular forces driving homogenization of rDNA repeats in concerted evolution.

## Introduction

The plant ribosomal DNA (rDNA) consists of highly conserved regions coding for 18S–5.8S–25S ribosomal RNAs (rRNAs), 45S rDNA, and 5S rRNAs, 5S rDNA, intertwined with more rapidly evolving nontranscribed spacer (NTS) sequences. Based on their high copy number in plant genomes, and structural features, the 45S rDNA and 5S rDNA loci have been broadly used in studies of plant systematics, evolution, and biodiversity and as molecular markers of ancestral genomes in polyploids and various hybrids (Borisjuk et al., 1988; Stadler et al., 1995; Mahelka et al., 2017). The 5S rDNA is especially well suited for such studies, due to the smaller size of its repeat units, making the sequences technically easier to handle compared to the much larger 45S rDNA, and to the higher variability exhibited by 5S rDNA NTSs relative to those of the 45S rDNA. The 5S rDNA loci have been characterized for representatives of numerous plant taxa to reveal phylogenetic relationships (Baker et al., 2000; Pan et al., 2000; Röser et al., 2001), genome evolution (Kellogg and Appels, 1995; Allaby and Brown, 2001; Simon et al., 2018), and the subgenome composition of polyploids (Kovarik et al., 2008; Baum and Feldman, 2010; Sergeeva et al., 2017; Volkov et al., 2017), natural hybrids, and artificial hybrids (Zanke et al., 1995; Fulnecek et al., 2002; Matyácek et al., 2002; Volkov et al., 2007; Mahelka et al., 2013).

Duckweeds are a group of floating plants present in local aquatic ecosystems worldwide, where they often cover large areas of the water surface (Landolt, 1986; Tippery and Les, 2020). Duckweeds were a favorite model for plant biochemistry studies from the 1950s to the 1980s before being supplanted by model plants, such as Arabidopsis (*Arabidopsis thaliana*). However, these aquatic plants came back into the spotlight in the 2010s, primarily because of their potential as a promising feedstock for the production of biofuels and other valuable biochemicals (Cao et al., 2018; Zhou and Borisjuk, 2019; Liu et al., 2021). Different duckweed species are also widely used for wastewater treatment (Zhao et al., 2015; Zhou et al., 2018) and biosensing (Ziegler et al., 2019). The establishment of living *in vitro* collections hosting ~2,000 duckweed ecotypes (Sree and Appenroth, 2020), primarily at the world duckweed depository hosted by Prof. E. Lam at the Rutgers Duckweed Stock Cooperative at Rutgers University, New Brunswick, NJ, United States,^1^ and a number of local collections in Canada, China, Germany, Hungary, India, Ireland, and Switzerland (Lam et al., 2020) have supported and helped promote modern duckweed research.

Duckweeds are an ancient group of monocot plants with extremely reduced morphology. Their exact taxonomic status, as a distinct family (Lemnaceae) or as a subfamily that belongs to the Araceae (Les and Tippery, 2013), is still debated (Tippery and Les, 2020). The 37 known species of duckweeds are currently classified into five genera: Spirodela, Landoltia, Lemna, Wolffia, and Wolffiella (Appenroth et al., 2013). The genus Landoltia, represented by the single species dotted duckweed or duckmeat (*Landoltia punctata*), is believed to have separated relatively recently from Spirodela, based on the morphological and new molecular data (Les and Crawford, 1999). In addition to the benefits commonly provided by duckweed species, such as fast, dense growth on the water surface, easy harvesting, convenient enzymatic saccharification of biomass, and efficient phytoremediation of wastewater (Xu and Shen, 2011; Zhao et al., 2015; Ziegler et al., 2015; Zhou et al., 2018), *L. punctata* has the added advantage of being one of the most resilient and stress-resistant among all duckweeds. For example, in the subtropical climate of Eastern China, *L. punctata* is the first duckweed species to colonize water reservoirs in the Spring and the last remaining in the Fall, thus exhibiting the longest vegetative growth period compared with other duckweeds. Based on these qualities, the species has attracted much attention as a promising, inexpensive, and sustainable source of valuable biomass for the production of biofuels, such as ethanol, butanol, biogas, and hydrogen (Tao et al., 2017; Toyama et al., 2018; Miranda et al., 2020), and high-value biochemicals, such as succinic acid (Shen et al., 2018).

In addition to these applications, the genetic diversity seen in duckweeds has stimulated a recent burst of studies examining duckweed genomics, molecular evolution, ecology, and biodiversity (Appenroth et al., 2015; Laird and Barks, 2018; Ho et al., 2019). Despite the fact that the duckweed 5S rDNA was one of the first sequenced genes in plants (Vandenberghe et al., 1984), the rDNA remains relatively poorly studied in duckweeds and in the Araceae. Our current knowledge of the molecular organization of the rDNA in the duckweed relates to the partial sequencing of 35S rDNA repeats from representative duckweed species in a study aiming to investigate the phylogenetic relationships and evolutionary history of Lemnaceae by Tippery et al. (2015) and to whole genome sequences of *Spirodela polyrhiza* (Michael et al., 2017) and *Spirodela intermedia* (Hoang et al., 2020), which revealed important characteristics of the 35S and 5S rDNA loci.

In this study, we present the molecular organization of the 5S rDNA locus in one *L. punctata* ecotype originating from Eastern China, based on the characterization of 25 independent sequences derived from cloned PCR products. Our results provide new information on the diversity and arrangement of the rDNA in this species and shed new light on general principles of evolution and arrangement of the 5S rDNA in plants.

## Materials and Methods

### Plant Materials

The duckweed ecotype used in this study was collected in summer of 2017 from a lake (GPS location: N 33″618817, E 119″001941) in one of the parks in the East China city of Huai’an. The collected fronds were surface sterilized in 0.5% sodium hypochlorite and 0.1% benzalkonium bromide in order to establish an aseptically grown strain. The NB0014 strain, developed from a single frond, is maintained as an *in vitro* culture on 0.8% agar containing 0.5× Schenk and Hildebrandt (SH) salts (Sigma-Aldrich, St. Louis, MO, United States) and 0.5% sucrose, pH 5.7–6.0, under axenic conditions. The identity of the NB0014 as the species of *Landoltia punctata* was confirmed by DNA barcoding using primers specific for chloroplast intergenic spacers atpF-atpH (ATP) and psbK-psbL (PSB), recommended by Consortium for the Barcode of Life (CBOL), as previously described (Borisjuk et al., 2015).

### Cloning and Sequence Characterization of *L. punctata* 5S rDNA Genes

For analysis of 5S rDNA genes, total DNA was isolated from the *in vitro* propagated biomass of *L. punctata* NB0014 using a cetyltrimethylammonium bromide (CTAB) method (Murray and Thompson, 1980) modified according to Borisjuk et al. (2015). The 5S rDNA genes were amplified from genomic DNA by PCR using the 5S rDNA gene-specific primers DW-5S-F: CTTGGGCGAGAGTAGTACTAGG and DW-5S-R: CACGCTTAACTTCGGAGTTCTG. The generated DNA fragments were purified by gel electrophoresis, cloned into the vector pMD19 (TaKaRa, Dalian, China), and custom sequenced by the Sangon Biotech (Shanghai, China). The obtained sequences were primarily analyzed using the “Online Analysis Tools” package.^2^ The subrepeats were characterized using the advanced hidden Markov model with the CLC Main Workbench (Version 6.9.2, QIAGEN Digital Insights, Redwood City, CA, United States) software. For the detection of the DNA regions likely to fold into G-quadruplex structures, we have primarily used the pqsfinder prediction tool (Labudová et al., 2020) available at the website: https://pqsfinder.fi.muni.cz/, with further verification by the G4Hunter algorithm (Brázda et al., 2019), freely available at the DNA Analyzer server: https://bioinformatics.ibp.cz.

### Estimation of 5S and 35S rDNA Copy Number

The estimation of 5S and 35S rDNA gene copies was carried out by quantitative PCR (qPCR), relating the rates of the DNA amplification of samples to the standard curve. The standard curves were established based on the amplification reads of independent dilution series of two specially constructed reference plasmids, pAS-Lp1 and pAS-Lp2. The plasmids were assembled using a backbone of pAS-Sp1, previously constructed to calculate rDNA copy number in *S. intermedia* (Hoang et al., 2020) by replacing a single-copy Actin gene specific for Spirodela with *L. punctata* sequences encoding nitrate reductase, NR (pAS-Lp1) and nitrite reductase, NiR (pAS-Lp2). The NR and NiR sequences were PCR amplified from genomic DNA of *L. punctata* using primers designed according to the gene sequences kindly shared by Todd Michael (J. Craig Venter Institute, San Diego, CA, United States). The arrangements of reference genes in pAS-Lp1 and pAS-Lp2 plasmids with primers used in qPCR are represented in Supplementary Figure S1. The number of rDNA gene copies was determined in qPCRs prepared with the UltraSybr Mixture (CWBio, Taizhou, China), run on the CFX Connect Real-Time Detection System (Bio-Rad, Hercules, CA, United States). The samples and 10-fold dilution series of the reference plasmids were assayed in the same run. The quality of products was checked by the thermal denaturation cycle. Only the experiments providing a single peak were considered. Three technical replicates were performed for each sample. The obtained data were analyzed using the program BIO-RAD CFX Manager 3.1 (Hercules, CA, United States) and Microsoft Excel 2016 software (Microsoft Corp., Redmond, WA, United States).

## Results

### Characterization of the 5S rDNA in *L. punctata*

**Figure 1:**
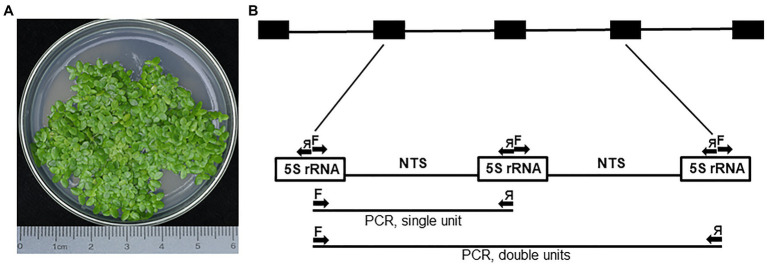
The PCR amplification of 5S rDNA units in *Landoltia punctata* NB0014. **(A)** Representative image of the *Landoltia punctata* ecotype NB0014 during *in vitro* culture; the species identity was confirmed by the chloroplast DNA barcoding. **(B)** Schematic representation of the complete 5S rDNA locus and the PCR amplification scheme. Arrows represent forward (F) and reverse (я) primers used during PCR and may amplify single or double 5S rRNA units. The array of black boxes at the top represents the 5S rDNA locus, and the white boxes represent the DNA sequences transcribed to produce the 5S rRNA. NTS, nontranscribed spacer.

*Landoltia punctata*, a duckweed species inhabiting mostly tropical and subtropical regions (Tippery and Les, 2020), is represented in this study by ecotype NB0014, which was isolated in the Jiangsu province of Eastern China. To detect possible intragenomic variation, we analyzed 5S rDNA repeats by cloning PCR products amplified with primers designed to cover the two halves of neighboring 5S rDNA genes with the NTS in the center, then sequencing individual clones. In total, 25 clones containing inserts ranging from 260 to 653 bp were sequenced and analyzed, including five clones containing two sequential 5S rDNA units (Figure 1).

### Conserved 5S rDNA Gene Sequence and rDNA Copy Number

All sequenced clones representing building blocks of the 5S rDNA locus consisted of a common unit of 119 bp coding for 5S rDNA and an adjacent NTS. Across the 30 5S rDNA sequences (from 20 clones with one copy and five clones with two copies of the rDNA), we detected six nucleotide substitutions but no variation in the 5S rDNA gene length (Supplementary Figure S2). Five of these variants were T/C or A/G transitions, with the final substitution being a T/G transversion.

**Figure 2:**
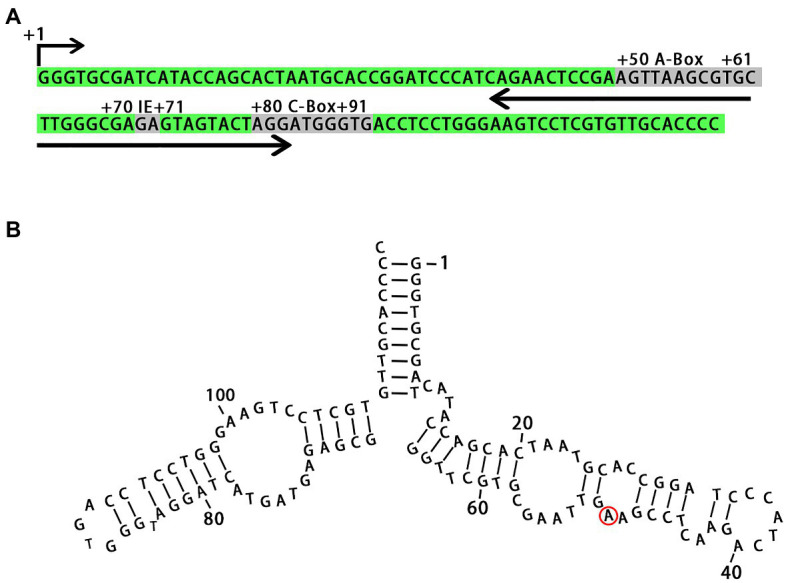
Primary nucleotide sequence of the 5S rDNA gene and secondary structure of the 5S rRNA. **(A)** Conserved motifs involved in transcriptional regulation are marked as A-box, IE, and C-box. **(B)** The position of nucleotide substitution specific for clones with short NTS (NB0014-22 and NB0014-23) is marked with a red ring. IE, intermediate element.

We identified all regulatory sequences in the 5S rDNA locus from *L. punctata*, such as the A-box, intermediate element (IE), and C-box, which are characteristic of plant genes (Hemleben and Werts, 1988; Cloix et al., 2003). The 5S rDNA transcribed from the locus was predicted to form a secondary structure similar to that seen in other plant species (Figure 2). The specific A/G transitions at nucleotide +50 in clones NB0014-22 and NB0014-23, which had the two shortest rDNA units with an NTS of 151 bp, mapped to a loop in the predicted 5S rDNA secondary structure, where it is unlikely to interfere with rDNA folding (Figure 2).

We next estimated the 5S rDNA and 25S rDNA gene copy numbers by qPCR using the approach previously developed to estimate rDNA copy number in *S. intermedia* (Hoang et al., 2020), with NR and NiR as two single-copy genes in *L. punctata* for normalization. We determined that the 5S rDNA locus was represented by 168 ± 25 gene copies, whereas the 35S rDNA had 176 ± 37 copies in the genome of the *L. punctata* ecotype NB0014.

### The NTS Shows Variant Subrepeat Structures

The 5S rDNA NTS region showed a significant variation in sequence length, ranging from 151 to 524 bp. A full alignment of the 30 NTS sequences represented in the 25 clones clearly separated them into two groups: one with 28 NTS sequences, and the other represented by two identical sequences from clones NB0014-16A and NB0014-25 (Supplementary Figure S3). Both classes were characterized by specific conserved sequences at their 5' ends (with lengths of 95 bp for NTS class I and 169 bp for NTS class II) and started with the transcription termination sequence TTTT (Hemleben and Werts, 1988). Both classes also shared a more variable TC-rich region in their center. In 26 out of the 28 clones with a class I NTS, the length of the NTS was over 400 bp, with a range between 432 and 524 bp (Supplementary Figure S4). The two remaining clones, NB0014-22 and NB0014-23, had the shortest variants, as they lacked a large portion of the 3' end, with the exception of the 12 nucleotides directly adjacent to the 5S rDNA gene (Supplementary Figure S5).

**Figure 3:**
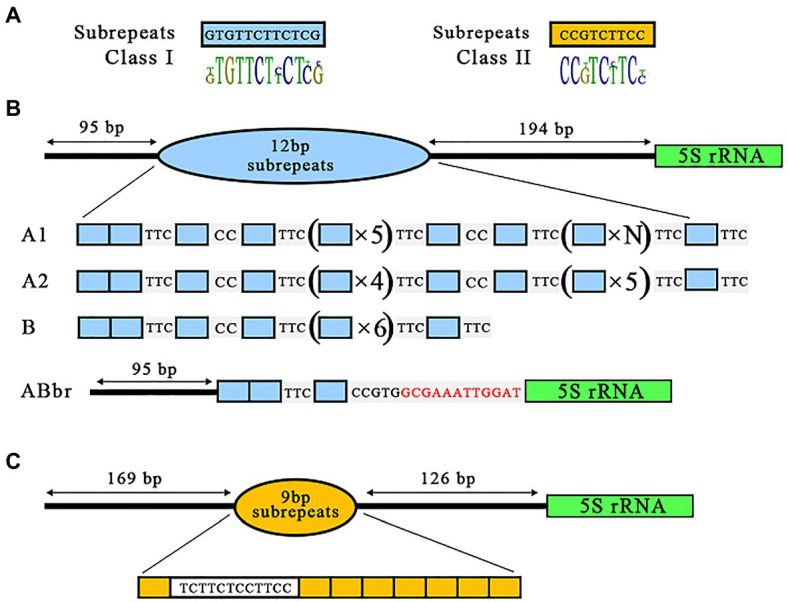
Diversity of 5S rDNA NTS repeats in the gene of *Landoltia punctata* NB0014. **(A)** The NB0014 5S rDNA NTS repeats are classified into class I and class II, based on the type, number, and arrangement of their 12-bp (blue blocks) or 9-bp (yellow blocks) subrepeats in relation to the 5S rDNA gene (marked by green; **B**). Class I is further divided into four subclasses: A1, A2, B, and ABbr. Red letters in the ABbr variant indicate the 12 nucleotides upstream of the 5S rDNA gene that are shared by all NTS class I sequences **(C)**. Class II is represented by a single NTS type of 9-bp units.

An in-depth analysis of all NTS sequences using CLC software (Version 6.9.2, QIAGEN Digital Insights, Redwood City, CA, United States) revealed that the variable central region of both NTS classes is composed of repeated units (subrepeats) of 12 or 9 bp for class I and class II NTS, respectively, in various arrangements. Notably, both NTS classes shared a 6-bp core (T-C-T/C-T/C-T/C-T/C; Figure 3). The 9-bp repeats detected in the two clones with class II NTS were organized as a single copy followed by a 12-bp sequence resembling an almost perfectly duplicated 6-bp core, tCTTCT-cCTTCC, followed by seven copies of the 9-bp element (Figure 3B).

The arrangements of the 12-bp units among clones with class I NTS were more complex and were divided into four subclasses (Figure 3A). Subclasses A1, A2, and B, represented by 26 sequences of class I NTS, all started with two copies of the 12-bp unit, followed by the sequence TCC, another 12-bp repeat, the sequence CC, one more 12-bp repeat and the sequence TCC. The three subclasses then diverged in their arrangement, with five (A1), four (A2), or six (B) 12-bp repeats. The predominant subclass A1 with 22 NTS clones was further extended by a block of four to six consecutive 12-bp units with no spacer sequence. Subclass A2, which was represented by three individual clones, showed a slight, distinct extension of its repeat sequence by a block of five consecutive 12-bp units. Finally, subclass B, represented by a single NTS variant, contained a single 12-bp unit following the basic unit described above. Subclasses A1, A2, and B were characterized by a highly conserved 194-bp sequence between the subrepeats and the 5S rDNA gene. We named the fourth subclass ABbr (for abbreviated), as it’s NTS comprised only three 12-bp repeats identical to subclasses A and B, but lacked the rest of the NTS sequences, with the exception of the final 12 bp upstream of the 5S rDNA gene (Figure 3B).

### Possible Alternative Regulation of 5S rDNA Variants in *L. punctata*

**Figure 4:**
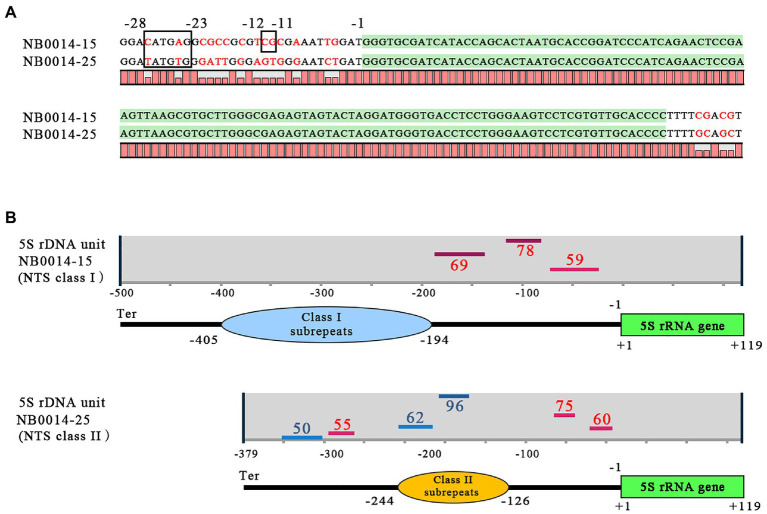
Features of class I and class II NTS sequences with the potential to modulate 5S rDNA transcription. **(A)** Regulatory DNA elements upstream and downstream of the 5S rDNA gene. The coding sequence for the 5S rRNA is highlighted in green; nucleotide positions from −28 to −23 mark the TATA-like motif; nucleotide positions −12 to −11 mark the GC dinucleotide; position −1 marks the first nucleotide upstream of the 5S rDNA transcription start. **(B)** Patterns of G-quadruplex structures predicted for class I and class II NTS sequences. Positions of G-quadruplex structures are indicated by horizontal lines (red for forward DNA strand, blue for reverse strand); numbers next to bars indicate the relative strength of each G-quadruplex structure, and the negative (−) numbers indicate their positions relative to the transcription start of the 5S rDNA gene; Ter marks the position of the terminator with sequence TTTT. NTS, nontranscribed spacer.

The basic regulatory elements for transcription by Pol III are located within the 5S rDNA gene sequence (Figure 1), but upstream cis-elements, such as TATA-like motifs and GC dinucleotides, may also significantly contribute to the modulation of transcription. The upstream regions of representative class I and class II NTSs of the 5S rDNA locus in *L. punctata* showed some divergence from the previously published arrangement in plants (Venkateswarlu et al., 1991; Cloix et al., 2003). For example, in clone NB0014-15, the GC dinucleotide was preserved at conserved position −12 and −11 from the transcription start in the class I 5S rDNA; however, the −28 to −23 location normally occupied by the TATATA-box in plants had the sequence ACATGA instead (Figure 4A). In the class II NTS 5S rDNA locus, represented by clone NB0014-25, the TATA-box was replaced by the related sequence ATATGT, but a TG dinucleotide occupied the −12 to −11 position, instead of the conserved GC dinucleotide.

We then used the pqsfinder algorithm (Labudová et al., 2020) to determine the potential for forming regulatory G-quadruplex structures (G4), which consist of four guanine-rich regions held together *via* unconventional base pairing (Lipps and Rhodes, 2009; Havlová and Fajkus, 2020). We discovered that the TG dinucleotide is a part of the 3' end of the sequence TGGGA on the reverse strand within the −37 to −8 sequence predicted to form a G4 structure, just upstream of the 5S rDNA gene (Figure 4B).

In addition to the differences in nucleotide organization noted above in the immediate 5S rDNA gene upstream regions, the general patterns of the revealed G-quadruplex structure were also distinct between class I and II NTSs. The pqsfinder algorithm predicted three G4-forming regions on the forward DNA strand of class I NTS, between the subrepeats and the 5S rDNA gene, with the highest score at position −142 to −106. Class II NTS had six potential G4-forming regions, three each on the forward and reverse DNA strand, scattered over the entire length of the NTS, with the highest scores obtained for the element located on the reverse strand within the subrepeats region. We validated these specific patterns with the G4 prediction tool G4Hunter (Brázda et al., 2019).

### Clones With Two 5S rDNA Units Hint at Low Repeat Homogenization Within the Locus

**Figure 5:**
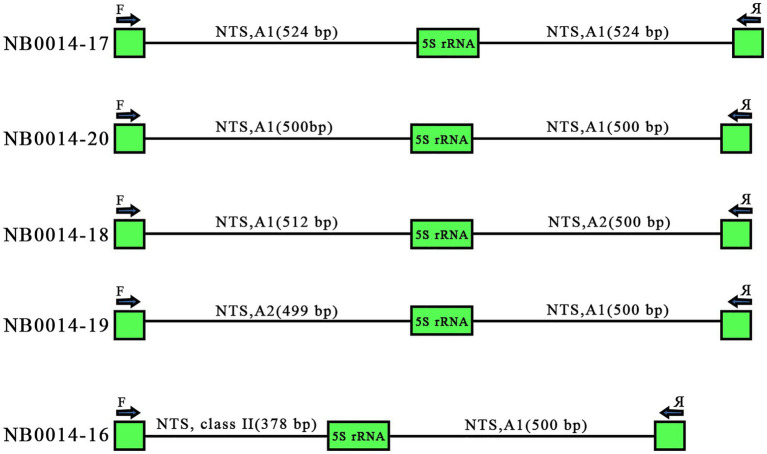
Various modes of 5S rDNA unit arrangement in clones containing pairs of consecutive repeated units. Clones NB0014-17 and NB0014-20 each contain identical A1 type NTS sequences of 524 bp and 500 bp, respectively. Clones NB0014-18 and NB0014-19 have interspersed type A1 and A2 units, whereas the clone NB0014-16 has interspersed class II and class I, type A1 units. NTS, nontranscribed spacer.

Tandemly repeated rDNA units are thought to undergo high levels of homogenization within the array, due to concerted evolution (Coen et al., 1982; Cronn et al., 1996). Of the 25 sequenced clones, five harbored two repeat units, as illustrated in Figure 1. These paired repeats offered us a glimpse into the arrangement of individual units along the 5S rDNA locus. Two clones with two 5S rDNA copies, NB0014-17 and NB0014-20, contained identical A1 variants of the NTS: two NTSs of 524 bp for NB0014-17 and two NTSs of 500 bp for NB0014-20. Three other clones harbored pairs of NTS each belonging to different classes, with NB0014-18 and NB0014-19 having class A1 and class A2 units. Even more surprising was the NTS pair in clone NB0014-16, with a class I type A1 unit followed by a class II NTS (Figure 5). The obtained results suggest a rather random mosaic arrangement of 5S rDNA units in *L. punctata*.

## Discussion

The duckweed 5S rDNA locus was among the first plant loci whose sequence was determined in the 1980s (Vandenberghe et al., 1984). However, rDNA loci of duckweeds did not attract substantial attention until recently. Tippery et al. (2015) investigated the phylogenetic relationships and evolutionary history of Lemnaceae by sequencing different regions of duckweed 35S rDNA repeats. Genome surveys revealed an unusually low 35S rDNA copy number in the great duckweed *S. polyrhiza* relative to other plants (Michael et al., 2017). Later studies of duckweed genomes showed that 5S rDNA genes are present as two loci in *S. polyrhiza*, *S. intermedia*, and *L. punctata* (Hoang et al., 2018, 2019). The recent sequencing of two *S. intermedia* genomes (Hoang et al., 2020) was consistent with these observations, and indicated that each 5S rDNA locus was populated by distinct units with locus-specific NTS. The total number of 5S rDNA copies was estimated to be around 70 per genome in *S. intermedia*, which is the lowest number reported to date for plants, as rDNA copy numbers typically reach the thousands.

The estimated rDNA copy number in the *L. punctata* ecotype NB0014 was 168 ± 25 copies for 5S rDNA and 176 ± 37 copies for 25S rDNA. These numbers are consistent with those seen in *S. polyrhiza* and *S. intermedia* when normalized to genome size, as the *L. punctata* genome is roughly three times bigger than that of Spirodela (Hoang et al., 2019). It is worth noting that the almost equal copy number for 5S rDNA and 25S rDNA units is atypical for plants, where the evolution of 5S and 25S rDNA loci appears to follow different patterns (Mahelka et al., 2013; Volkov et al., 2017); rather, the *L. punctata* pattern is more reminiscent of animal genomes, including human (Gibbons et al., 2015).

The two major classes of 5S rDNA repeats detected in *L. punctata* are in agreement with the genomic data produced for *S. polyrhiza* and *S. intermedia*, both of which have two types of 5S rDNA units composed of a conserved 119-bp gene coding sequence interspersed with two types of NTS, with slight but significant sequence differences in *S. intermedia* (Hoang et al., 2020) and much more profound differences both in length (~400 vs. ~1,070 bp) and nucleotide composition in *S. polyrhiza*, as can be seen in online databases for strain 7498^3^ and strain 9509.^4^ However, there were significant differences between the arrangements of the 5S rDNA locus in the two Spirodela species and *L. punctata*. First, almost no intragenomic or intergenomic variation was observed within each NTS type in Spirodela, which is in agreement with our own analysis of a smaller NTS variant in four *S. polyrhiza* ecotypes (Borisjuk et al., 2018). By contrast, we identified extensive variation in class I NTS in *L. punctata*, as illustrated in Figure 3. Second, both class I and class II NTSs in *L. punctata* contained multiple subrepeats, which is typical for intergenic spacers of 25S rDNA (Borisjuk and Hemleben, 1993; Borisjuk et al., 1997; Hemleben et al., 2021) but generally not observed for plant 5S rDNA spacers, including Spirodela.

In addition, because variation in NTS sequences directly adjacent to the 5S rDNA gene may modulate transcription (Figure 4A), NTS subrepeats may also contribute to regulating rDNA activity in a manner similar to 25S rDNA (Zentgraf and Hemleben, 1992; Schlögelhofer et al., 2002). This assumption is further strengthened by a prediction of strong 4G structures, which participate in the regulation of gene expression in many eukaryotic organisms (Lipps and Rhodes, 2009; Yadav et al., 2017), in the subrepeat region of class II *L. punctata* NTS (Figure 4B).

Even more intriguing was the finding that in three out of five clones with PCR amplicons composed of double 5S rDNA units, each neighboring repeat was represented by a distinct type of NTS (Figure 5). This result contradicts the basic concept of extended repeat homogenization along rDNA arrays (Eickbush and Eickbush, 2007) and contrasts with the arrangement of the 5S rDNA locus in the related Spirodela species, where each locus contained a single type of 5S rDNA unit, as shown by extra-long OxfordNano sequencing.^5^

Thus our finding, coupled with a recent discovery of variation and clustering of 35S rDNA repeats within the nucleolus organizing region (NOR2) locus in Arabidopsis (Sims et al., 2021), raises a question of the extent of homogeneity for rDNA repeats within their loci. Is the level of repeat homogenization species-specific, and if so, what are the mechanisms responsible for the differential manifestation of concerted evolution?

## Conclusion

Our data provide the first comprehensive report on the arrangement of 5S rDNA in a representative of the Araceae plant family. In particular, the study reveals two major classes of repeated units, which differ by the composition and distribution of subrepeats in the nontranscribed intergenic spacer of the 5S rDNA, and representations of DNA elements potentially involved in the regulation of 5S rDNA transcription. The genome of *L. punctata* has one of the lowest copy numbers of rDNA genes among flowering plants and an unusual, mosaic arrangement of 5S rDNA clusters. Overall, the findings of our study shed a new light on the organization of plant rDNA and may stimulate further discussion of plant genome evolution and the molecular forces that drive the homogenization of rDNA repeats.

## Data Availability Statement

The datasets presented in this study can be found in online repositories. The names of the repository/repositories and accession number(s) can be found in the article/Supplementary Material.

## Author Contributions

GC generated the sequencing data and prepared the manuscript’s figures. AS conducted genes quantifications by qPCR, organized and analyzed the data. NB conceived the idea and prepared the manuscript with contributions from GC and AS. All authors reviewed and approved the final manuscript.

### Conflict of Interest

The authors declare that the research was conducted in the absence of any commercial or financial relationships that could be construed as a potential conflict of interest.

## Footnotes

^1^http://www.ruduckweed.org

^2^https://molbiol-tools.ca

^3^https://genomevolution.org/coge/GenomeInfo.pl?gid=55812

^4^https://genomevolution.org/coge/GenomeInfo.pl?gid=51364

^5^https://genomevolution.org

## Supplementary Material

Supplementary Figure S1 Schematic representation of pAS-Lp1 and pAS-Lp-2 plasmids containing sequences of 25S rDNA (yellow), 5S rDNA (green), and the sequences of single copy genes for nitrate reductase (NR; dark blue) and nitrite reductase (NiR; violet). The specific primer pairs to amplify each reference gene are as follows: 5'-TCCCACTGTCCCTGTCTACT/5'-CCCACTTATCCTACACCTCT for 25S rDNA; 5'-GGGTGCGATCATACCAGCAC/5'-GGGTGCAACACGAGGACTTC and 5'-GGGTGCGATCATACCAGCAC/5'-GGGTGCAACACGAGGACTTC for 5S rDNA; 5'-ATTTTCCTTTCCGCCACC/5'-CCGATGTACTCAATGTGCC for NR; and 5'-CGCCAAAAGACGCGCATGA/5'-AAGACCGATGAAGCAGAACCC for NiR. The GenBank accession numbers are: pAS-Lp1, MW803142; pAS-Lp2, MW841295.

Supplementary Figure S2 Nucleotide alignment of 30 sequences of the 5S rDNA genes cloned from *Landoltia punctata*. Most of the aligned sequences are compilations of the halves of neighboring gene sequences, the sequences marked with M are the 5S genes in the middle of clones containing two consecutive 5S rDNA units, as depicted in Figure 1B. The randomly varied nucleotides are highlighted by blue; the nucleotide substitution specific for the clones with short NTS (NB0014-22 and NB0014-23) at position 50, are highlighted by violet. NTS, nontranscribed spacer.

Supplementary Figure S3 Bulk alignment of *Landoltia punctata* 5S rDNA NTS sequences. The sequences embrace the whole intergenic region between two 5S rDNA genes. The sequence with their names followed by A and B represent the two NTS from clones containing two consecutive 5S rDNA repeats according to Figure 1B. Sequence areas which differ from the consensus are highlighted in blue.

Supplementary Figure S4 Nucleotide alignment of the *Landoltia punctata* 5S rDNA NTS class I sequences. 12 bp subrepeats are marked by black rectangles. Sequence areas that differ from the consensus are highlighted in blue.

Supplementary Figure S5 Nucleotide alignment of the *Landoltia punctata* 5S rDNA NTS class II sequences. 9 bp subrepeats are marked by black rectangles.
