# Supplementary material for: Mosaic Arrangement of the 5S rDNA in the Aquatic Plant Landoltia punctata (Lemnaceae)
Source: Front Plant Sci. 2021 Jun 24;12:678689. doi: 10.3389/fpls.2021.678689 (PMC8264772; doi:10.3389/fpls.2021.678689)
Supplement: Supplementary file 1 [file Data_Sheet_1.zip › Supplementary Material/FigureS2.docx]

**Supplementary Figure S2.**

**50**

**l**

**NB0014-1 GGGTGCGATCATACCAGCACTAATGCACCGGATCCCATCAGAACTCCGAAGTTAAGCGTG -60**

**NB0014-2 GGGTGCGATCATACCAGCACTAATGCACCGGATCCCATCAGAACTCCGAAGTTAAGCGTG**

**NB0014-3 GGGTGCGATCATACCAGCACTAATGCACCGGATCCCATCAGAACTCCGAAGTTAAGCGTG**

**NB0014-4 GGGTGCGATCATACCAGCACTAATGCACCGGATCCCATCAGAACTCCGAAGTTAAGCGTG**

**NB0014-5 GGGTGCGATCATACCAGCACTAATGCACCGGATCCCATCAGAACTCCGAAGTTAAGCGTG**

**NB0014-6 GGGTGCGATCATACCAGCACTAATGCACCGGATCCCATCAGAACTCCGAAGTTAAGCGTG**

**NB0014-7 GGGTGCGATCATACCAGCACTAATGCACCGGATCCCATCAGAACTCCGAAGTTAAGCGTG**

**NB0014-8 GGGTGCGATCATACCAGCACTAATGCACCGGATCCCATCAGAACTCCGAAGTTAAGCGTG**

**NB0014-9 GGGTGCGATCATACCAGCACTAATGCACCGGATCCCATCAGAACTCCGAAGTTAAGCGTG**

**NB0014-10 GGGTGCGATCATACCAGCACTAATGCACCGGATCCCATCAGAACTCCGAAGTTAAGCGTG**

**NB0014-11 GGGTGCGATCATACCAGCACTAATGCACCGGATCCCAGCAGAACTCCGAAGTTAAGCGTG**

**NB0014-12 GGGTGCGATCATACCAGCACTAATGCACCGGATCCCATCAGAACTCCGAAGTTAAGCGTG**

**NB0014-13 GGGTGCGATCATACCAGCACTAATGCACCGGATCCCATCAGAACTCCGAAGTTAAGCGTG**

**NB0014-14 GGGTGCGATCATACCAGCACTAATGCACCGGATCCCATCAGAACTCCGAAGTTAAGCGTG**

**NB0014-15 GGGTGCGATCATACCAGCACTAATGCACCGGATCCCATCAGAACTCCGAAGTTAAGCGTG**

**NB0014-16 GGGTGCGATCATACCAGCACTAATGCACCGGATCCCATCAGAACTCCGAAGTTAAGCGTG**

**NB0014-16M GGGTGCGATCATACCAGCACTAATGCACCGGATCCCATCAGAACTCCGAAGTTAAGCGTG**

**NB0014-17 GGGTGCGATCATACCAGCACTAATGCACCGGATCCCATCAGAACTCCGAAGTTAAGCGTG**

**NB0014-17M GGGTGCGATCATACCAGCACTAATGCACCGGATCCCATCAGAACTCCGAAGTTAAGCGTG**

**NB0014-18 GGGTGCGATCATACCAGCACTAATGCACCGGATCCCATCAGAACTCCGAAGTTAAGCGTG**

**NB0014-18M GGGTGCGATCATACCAGCACTAATGCACCGGATCCCATCAGAACTCCGAAGTTAAGCGTG**

**NB0014-19 GGGTGCGATCATACCAGCACTAATGCACCGGATCCCATCAGAACTCCGAAGTTAAGCGTG**

**NB0014-19M GGGTGCGATCATACCAGCACTAATGCACCGGATCCCATCAGAACTCCGAAGTTAAGCGTG**

**NB0014-20 GGGTGCGATCATACCAGCACTAATGCACCGGATCCCATCAGAACTCCGAAGTTAAGCGTG**

**NB0014-20M GGGTGCGATCATACCAGCACTAATGCACCGGATCCCATCAGAACTCCGAAGTTAAGCGTG**

**NB0014-21 GGGTGCGATCATACCAGCACTAATGCACCGGATCCCATCAGAACTCCGAAGTTAAGCGTG**

**NB0014-22 GGGTGCGATCATACCAGCACTAATGCACCGGATCCCATCAGAACTCCGAGGTTAAGCGTG**

**NB0014-23 GGGTGCGATCATACCAGCACTAATGCACCGGATCCCATCAGAACTCCGAGGTTAAGCGTG**

**NB0014-24 GGGTGCGATCATACCAGCACTAATGCACCGGATCCCATCAGAACTCCGAAGTTAAGCGTG**

**NB0014-25 GGGTGCGATCATACCAGCACTAATGCACCGGATCCCATCAGAACTCCGAAGTTAAGCGTG**

*************************************** ***********.************

**NB0014-1 CTTGGGCGAGAGTAGTACTAGGATGGGTGACCTCCTGGGAAGTCCTCGTGTTGCACCCC -119**

**NB0014-2 CTTGGGCGAGAGTAGTACTAGGGTGGGTGACCTCCTGGGAAGTCCTCGTGTTGCACCCC**

**NB0014-3 CTTGGGCGAGAGTAGTACTAGGGTGGGTGACCTCCTGGGAAGTCCTCGTGTTGCACCCC**

**NB0014-4 CTTGGGCGAGAGTAGTACTAGGATGGGTGACCTCCTGGGAAGTCCTCGTGTTGCACCCC**

**NB0014-5 CTTGGGCGAGAGTAGTACTAGGATGGGTGACCTCCTGGGAAGTCCTCGTGTTGCACCCC**

**NB0014-6 CTTGGGCGAGAGTAGTACTAGGATGGGTGACCTCCTGGGAAGTCCTCGTGTTGCACCCC**

**NB0014-7 CTTGGGCGAGAGTAGTACTAGGATGGGTGACCTCCTGGGAAGTCCTCGTGTTGCACCCC**

**NB0014-8 CTTGGGCGAGAGTAGTACTAGGATGGGTGACCTCCTGGGAAGTCCTCGTGTTGCACCCC**

**NB0014-9 CTTGGGCGAGAGTAGTACTAGGATGGGTGACCTCCTGGGAAGTCCTCGTGTTGCACCCC**

**NB0014-10 CTTGGGCGAGAGTAGTACTAGGATGGGTGACCTCCTGGGAAGTCCTCGTGTTGCACCCC**

**NB0014-11 CTTGGGCGAGAGTAGTACTAGGATGGGTGACCTCCTGGGAAGTCCTCGTGTTGCACCCC**

**NB0014-12 CTTGGGCGAGAGTAGTACTAGGATGGGTGACCTCCTGGGAAGTCCTCGTGTTGCACCCC**

**NB0014-13 CTTGGGCGAGAGTAGTACTAGGATGGGTGACCTCCTGGGAAGTCCTCGTGTTGCACCCC**

**NB0014-14 CTTGGGCGAGAGTAGTACTAGGATGGGTGACCTCCTGGGAAGTCCTCGTGTTGCACCCC**

**NB0014-15 CTTGGGCGAGAGTAGTACTAGGATGGGTGACCTCCTGGGAAGTCCTCGTGTTGCACCCC**

**NB0014-16 CTTGGGCGAGAGTAGTACTAGGATGGGTGACCTCCTGGGAAGTCCTCGTGTTGCACCCC**

**NB0014-16M CTTGGGCGAGAGTAGTACTAGGATGGGTGACCTCCTGGGAAGTCCTCGTGTTGCACCCC**

**NB0014-17 CTTGGGcGAGAGTAGTACTAGGATGGGTGACCTCCTGGGAAGTCCTCGTGTTGCACCCC**

**NB0014-17M CTTGGGCGAGAGTAGTACTAGGATGGGTGACCTCCTGGGAAGTCCTCGTGTTGCACCCC**

**NB0014-18 CTTGGGCGAGAGTAGTACTAGGATGGGTGACCTCCTGGGAAGTCCTCGTGTTGCACCCC**

**NB0014-18M CTTGGGCGAGAGTAGTACTAGGATGGGTGACCTCCTGGGAAGTCCTCGTGTTGCACCCC**

**NB0014-19 CTTGGGCGAGAGTAGTACTAGGATGGGTGACCTCCTGGGAAGTCCTCGTGTTGCACCCC**

**NB0014-19M CTTGGGCGAGAGTAGTACTAGGATGGGTGACCTCCTGGGAAGTCCTCGTGTTGCACCCC**

**NB0014-20 CTTGGGCGAGAGTAGTACTAGGATGGGTGACCTCCTGGGAAGTCCTCGTGTTGCACCCC**

**NB0014-20M CTTGGGCGAGAGTAGTACTAGGATGGGTGACCTCCTGGGAAGTCCTCGTGTTGCACCCC**

**NB0014-21 CTTGGGCGAGAGTAGTACTAGGATGGGTGACCTCCCGGGAAGTCCTCGTGTTGCACCCC**

**NB0014-22 CTTGGGCGAGAGTAGTACTAGGATGGGTGACCTCCTGGGAAGTCCTCGTGTTGCACCCC**

**NB0014-23 CTTGGGCGAGAGTAGTACTAGGATGGGTGACCTCCTGGGAAGTCCTCGTGTTGCACCCC**

**NB0014-24 CTTGGGCGAGAGTAGTACTAGGATGGGTGACCTCCTGGGAAGTCCTCGTGTTGCACCCC**

**NB0014-25 CTTGGGCGAGAGTAGTACTAGGATGGGTGACCTCCTGGGAAGTCCTCGTGTTGCACCCC**

************************.************.*************************
