# Supplementary figures and images for: Mosaic Arrangement of the 5S rDNA in the Aquatic Plant Landoltia punctata (Lemnaceae)
Source: Front Plant Sci. 2021 Jun 24;12:678689. doi: 10.3389/fpls.2021.678689 (PMC8264772; doi:10.3389/fpls.2021.678689)

## Slide 1
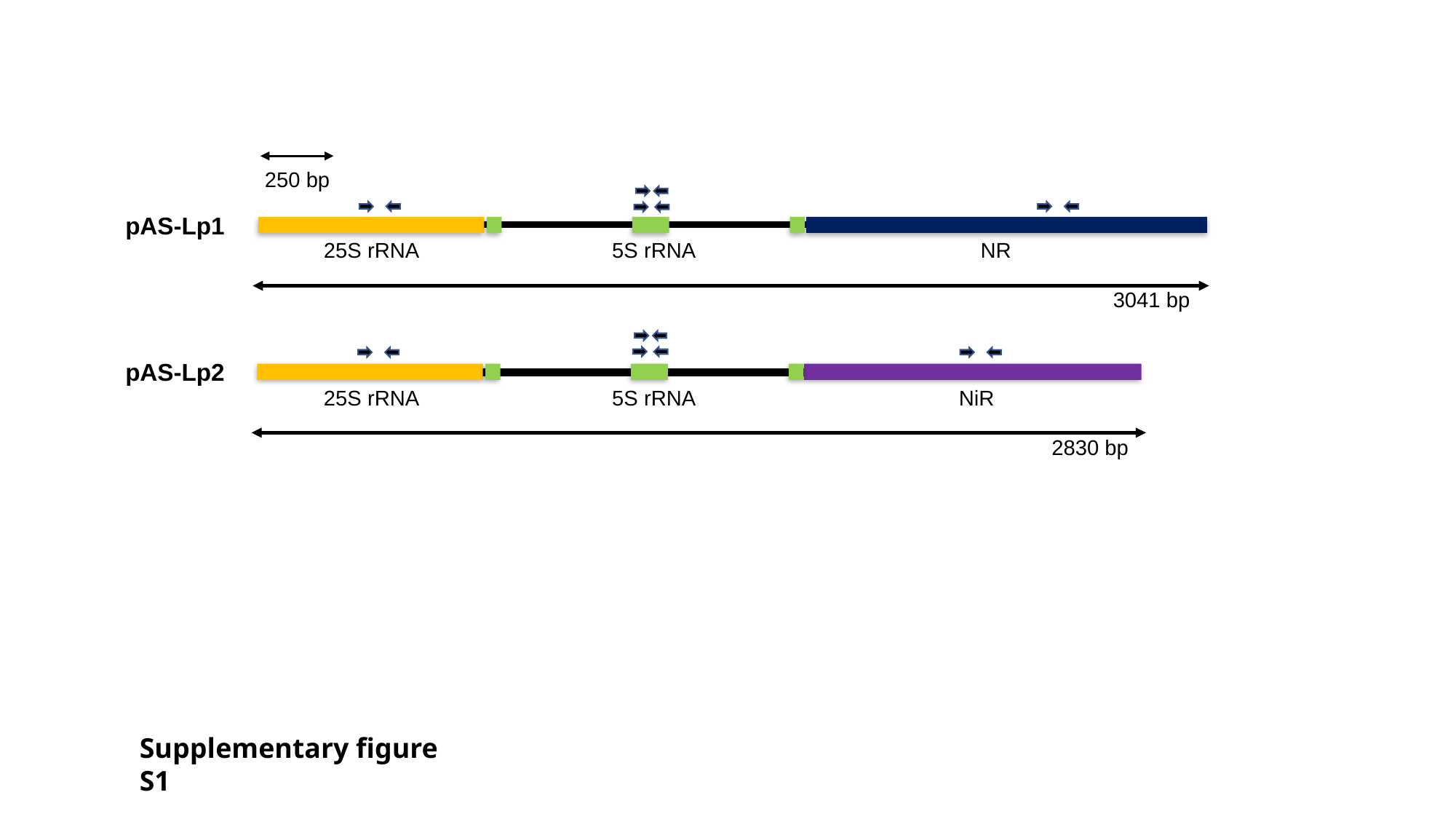

250 bp
pAS-Lp1
 25S rRNA
5S rRNA
NR
3041 bp
pAS-Lp2
 25S rRNA
5S rRNA
NiR
2830 bp
Supplementary figure S1

Supplement: Supplementary file 1 [file Data_Sheet_1.zip › Supplementary Material/FigureS1.PPTX]

**Supplementary Figure S3**.


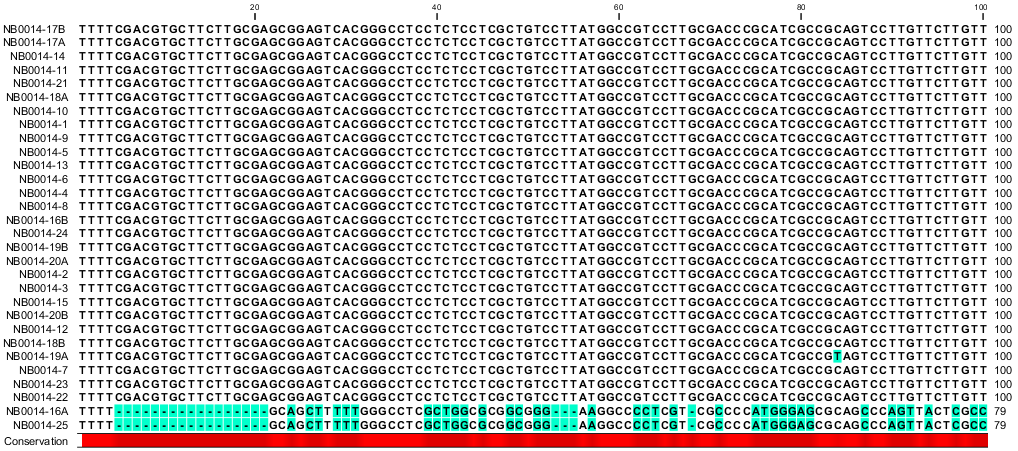


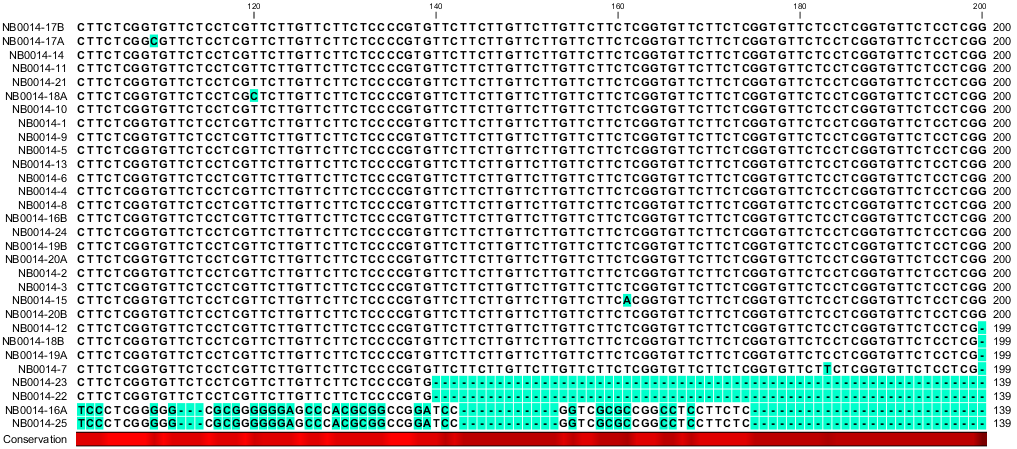


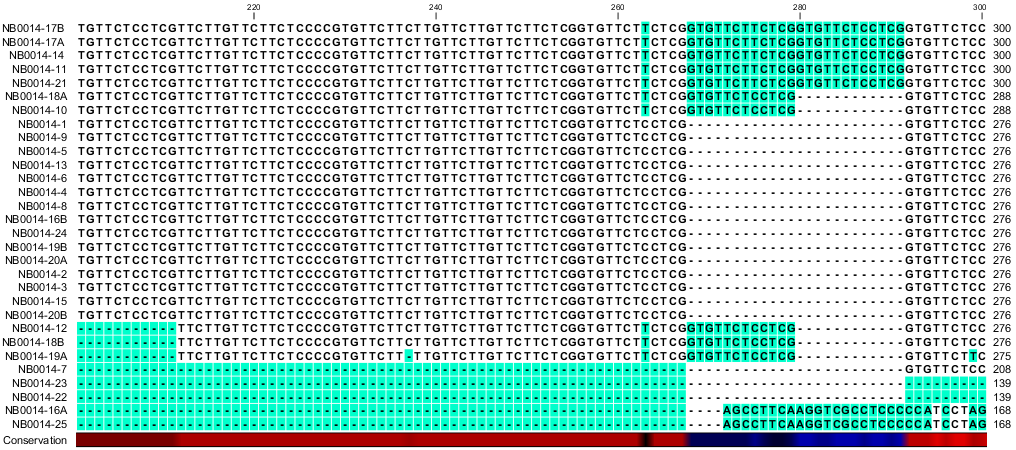


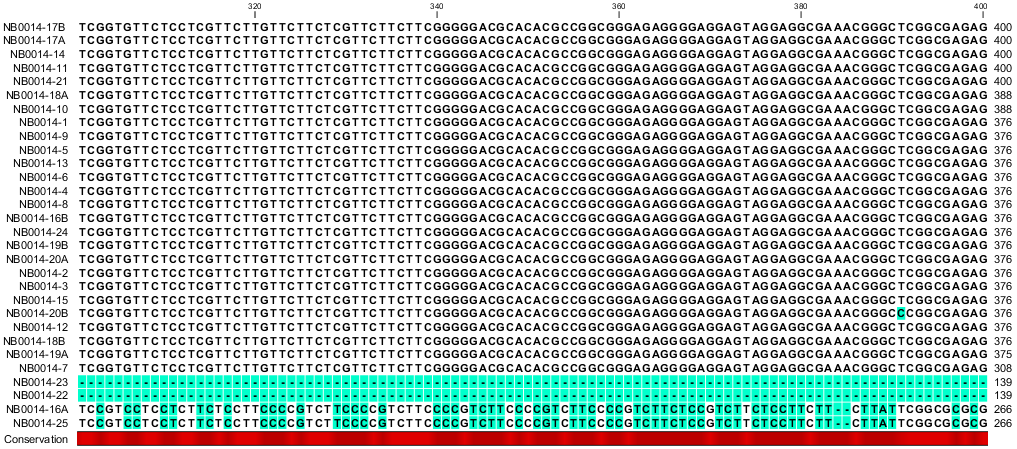


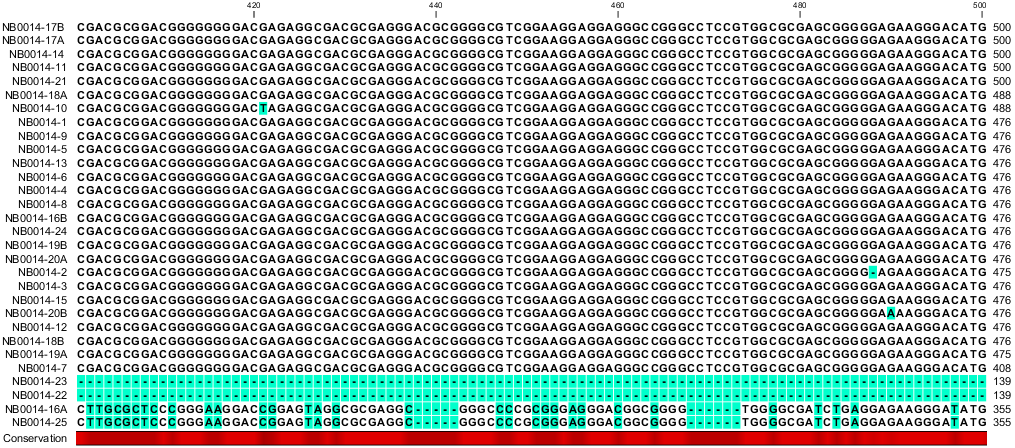


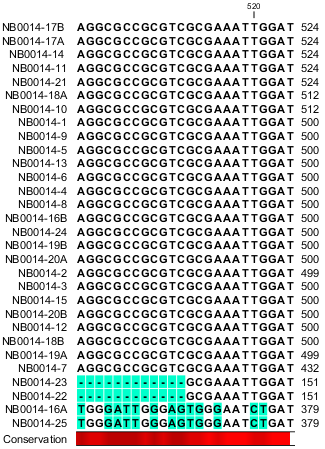

Supplement: Supplementary file 1 [file Data_Sheet_1.zip › Supplementary Material/FigureS3.docx]

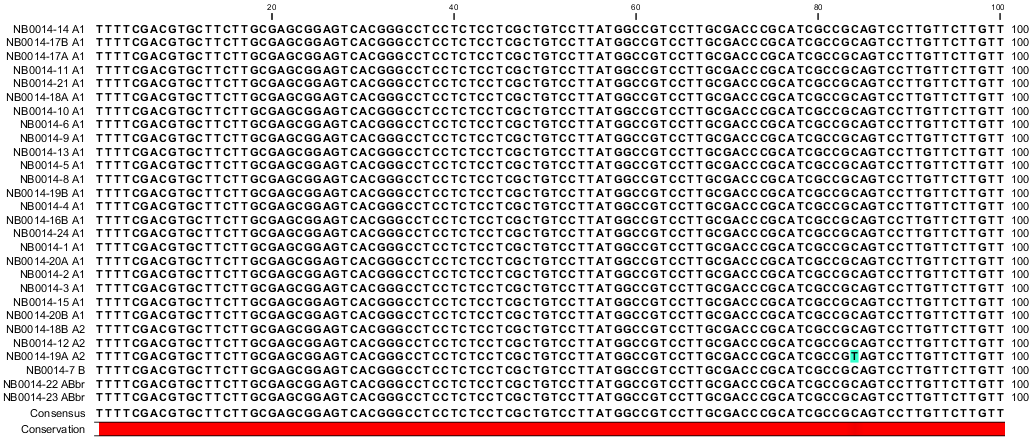

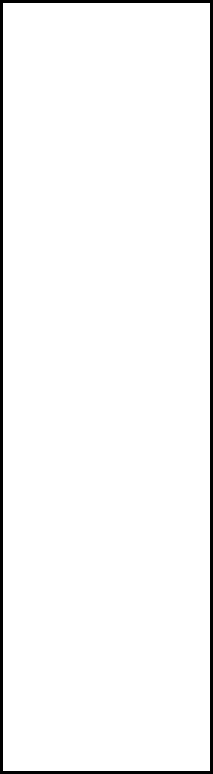


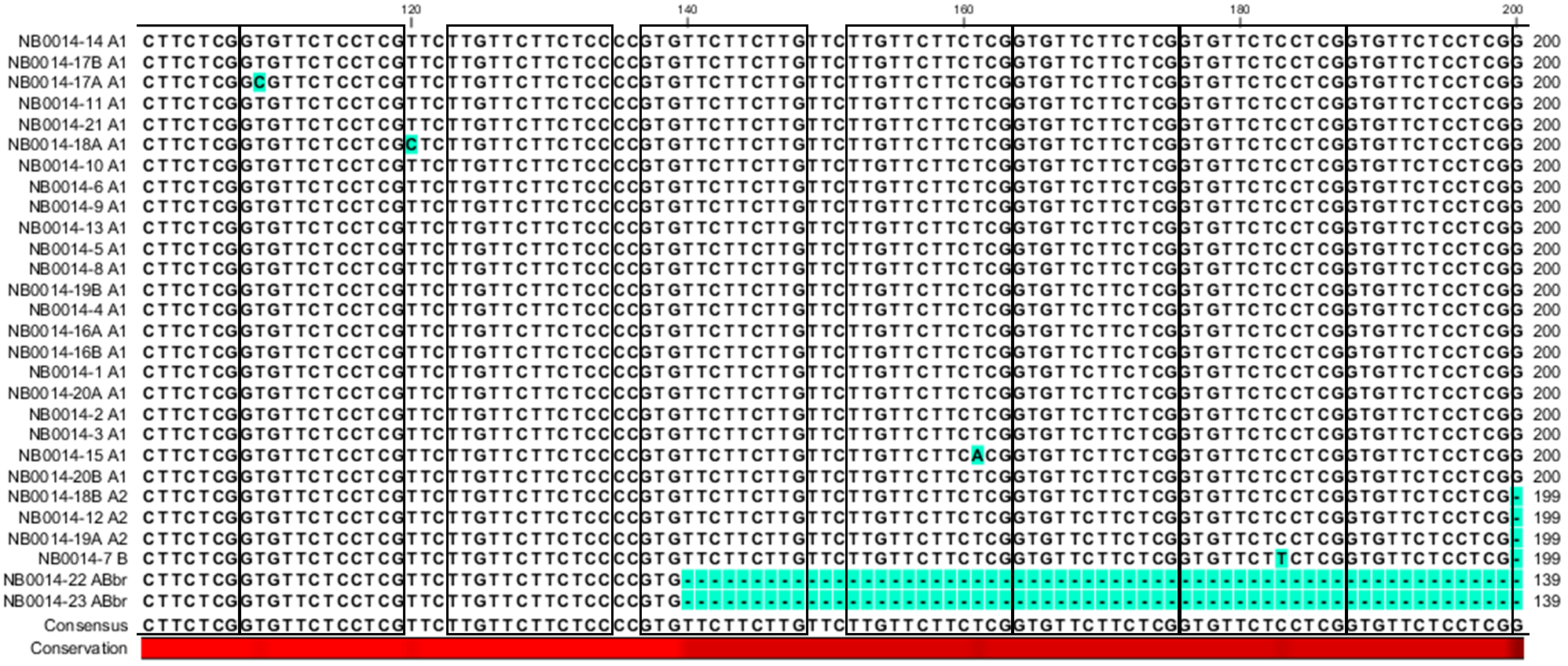


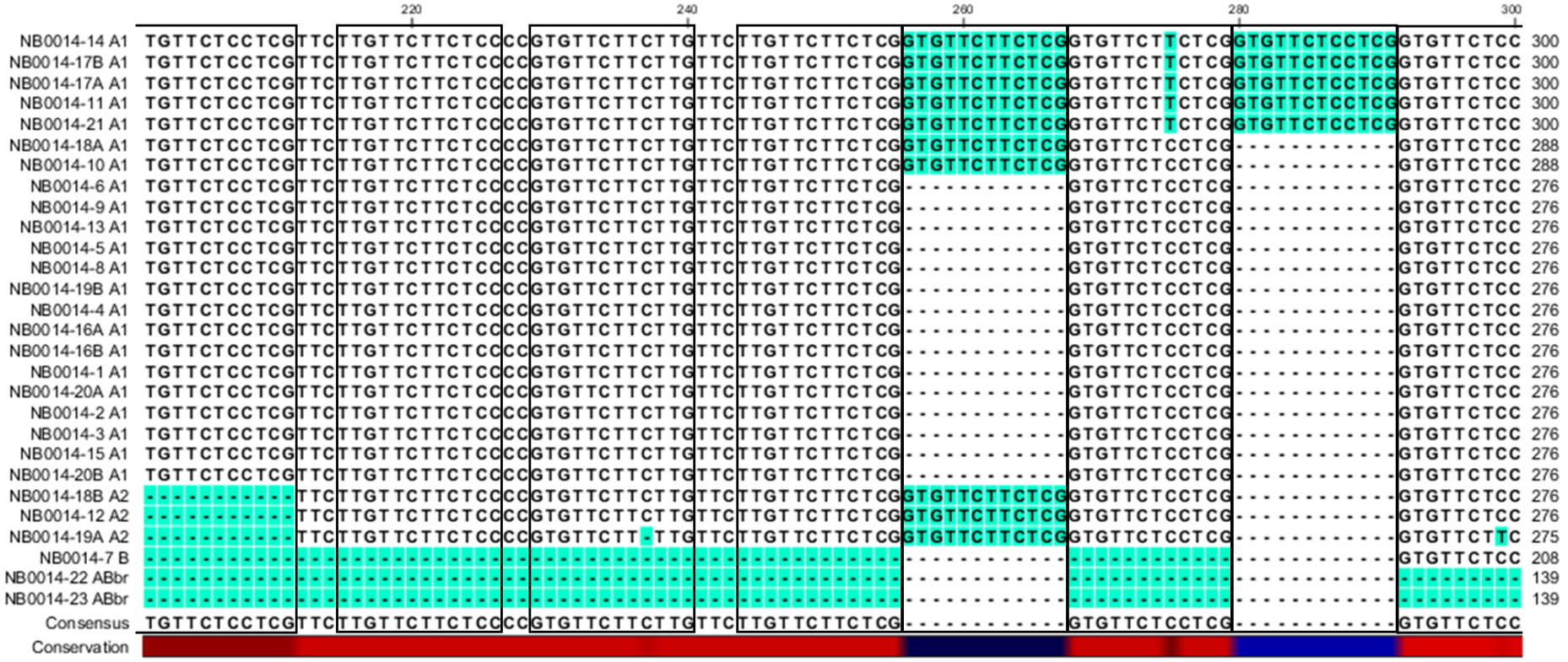


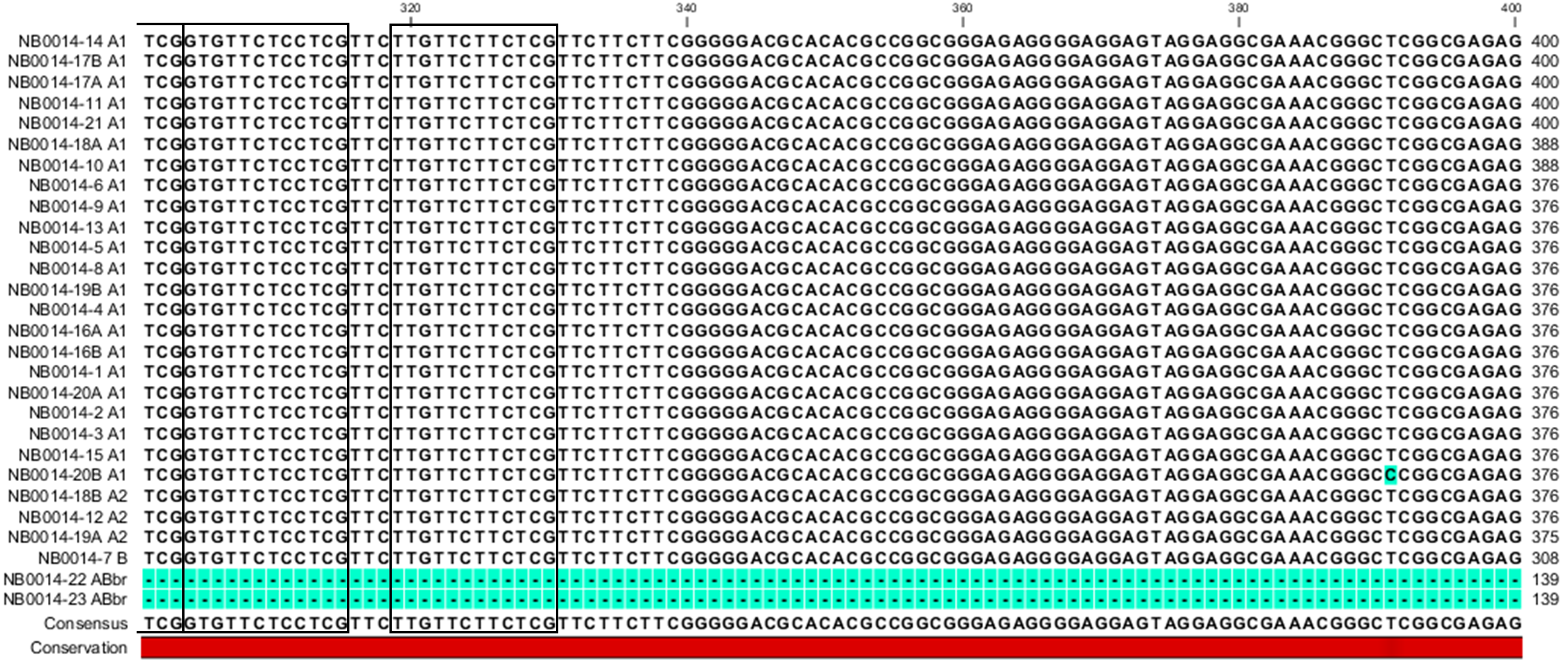


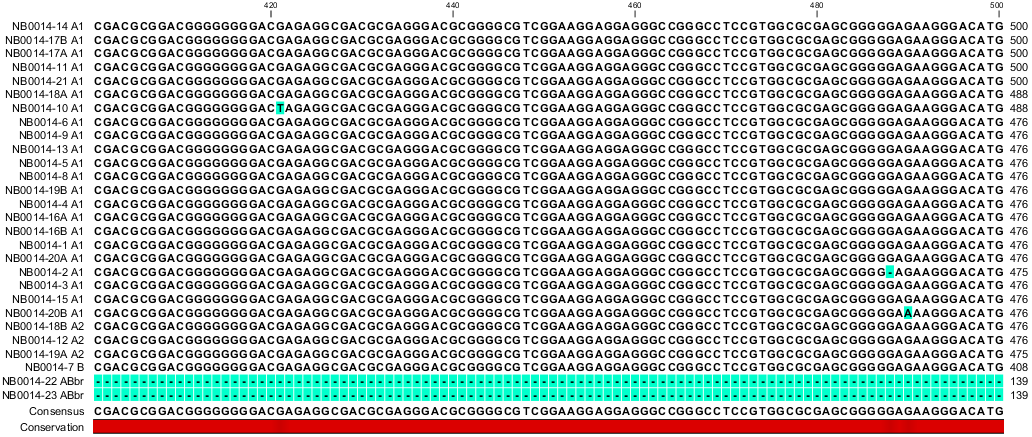


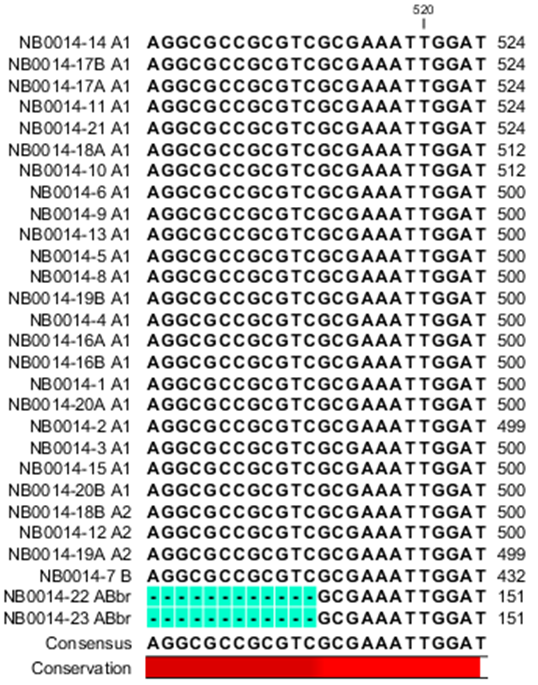

Supplement: Supplementary file 1 [file Data_Sheet_1.zip › Supplementary Material/FigureS4.docx]

**Supplementary Figure S5**.


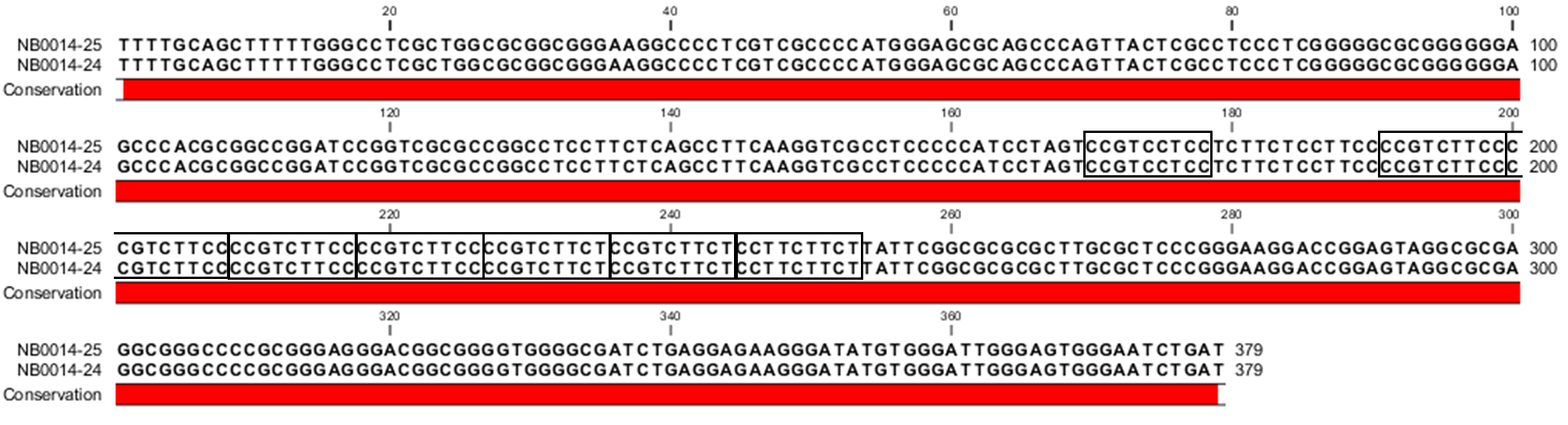

Supplement: Supplementary file 1 [file Data_Sheet_1.zip › Supplementary Material/FigureS5.docx]
